# Supplementary material for: Content-rich biological network constructed by mining PubMed abstracts
Source: BMC Bioinformatics. 2004 Oct 8;5:147. doi: 10.1186/1471-2105-5-147 (PMC528731; doi:10.1186/1471-2105-5-147)
Supplement: Additional File 2 — The original results of the above study (non-essential files are deleted to keep the file size under the limit set by BMC bioinformatics). [file 1471-2105-5-147-S2.bz2 › chilibotAdditionalFile2/dip20/404ID8955093E10064/html/LIFR_OSM.html]

 


 **LIFR** and **OSM** 
  
Found 43 abstracts in PubMed, retrieved 20.  
 

 What does Google say? 
 PDF only 
| .edu only 

---

**Interactive relationship** (e.g. stimulation, inhibition, etc)

**Inhibitory relationship**- The cytokine receptor subunits gp130, leukemia inhibitory factor receptor  [ **LIFR** ]  alpha LIFRalpha, and oncostatin M  [ **OSM** ]  receptor beta OSMRbeta transduce  **OSM**  signals that regulate gene expression and cell proliferation.  Ref: 11602599 J Biol Chem, 2001
- Oncostatin M  [ **OSM** ]  regulates the synthesis and turnover of gp130, leukemia inhibitory factor receptor  [ **LIFR** ]  alpha, and oncostatin M  [ **OSM** ]  receptor beta by distinct mechanisms.  Ref: 11602599 J Biol Chem, 2001
- Using neutralizing antibodies to gp 130, the  **OSM**  receptor OSMR, and the leukemia inhibitory factor receptor  [ **LIFR** ]   **LIFR** , we document that  **OSM**  exclusively uses the OSMR gp 130 heterodimer in signaling events, rather than the  **LIFR**  gp 130 heterodimer.  Ref: 10899931 J Neurochem, 2000
- The leukemia inhibitory factor LIF, cardiotrophin 1 CT 1 and oncostatin M  [ **OSM** ]   **OSM**  are four helix bundle cytokines acting through a common heterodimeric receptor composed of gp130 and LIF receptor  **LIFR** .  Ref: 12707269 J Biol Chem< MedlineTA>J Biol Chem, 2000
- The leukemia inhibitory factor receptor  [ **LIFR** ]  LIF R  [ **LIFR** ]  subunit is a component of cell surface receptor complexes for the multifunctional cytokines, LIF, cardiotrophin 1, ciliary neurotrophic factor, and human oncostatin M  [ **OSM** ] .  Ref: 11042511 Leuk Lymphoma, 2000
**Neutral relationship**- Both mouse and human  **OSM**  engaged gp130 with their respective OSMRbeta subunits, but only human  **OSM**  also acted through  **LIFR** .  Ref: 10854424 J Biol Chem, 2000
- The amount of  **LIFR**  was down regulated in both LIF and  **OSM**  producing cells and this effect was not found in wild type U373 MG cells treated with externally added cytokines.  Ref: 11284716 Biochem J, 2001
- Our results indicate that LIF, CT 1 and  **OSM**  share an overlapping binding site located in the Ig like domain of  **LIFR** .  Ref: 12707269 J Biol Chem< MedlineTA>J Biol Chem, 2001
- The different behavior of LIF and CT 1, on one side, and of  **OSM** , on the other side, can be related to the different affinity of their site III for  **LIFR** .  Ref: 12707269 J Biol Chem< MedlineTA>J Biol Chem, 2001
- Oncostatin M  [ **OSM** ]  can activate two related receptors, one consisting of a low affinity LIF receptor subunit,  **LIFR**  beta, which forms a heterocomplex with the gp130 signal transducing protein and can recognize both oncostatin M  [ **OSM** ]  and LIF, and a second heterocomplex consisting of a subunit that specifically recognizes oncostatin M  [ **OSM** ] , OSMR beta, and the gp130 protein.  Ref: 12061840 Cytokine, 2002
- Recombinant human Oncostatin M  [ **OSM** ]  rhOSM can induce the transient tyrosine 705 phosphorylation of STAT3 in serum starved  **LIFR**  beta OSMR beta expressing ovarian carcinoma cell lines, but does not alter cell growth and effects only a modest increase in the apoptotic rate in these cultured cells.  Ref: 12061840 Cytokine, 2002

**Non-interactive relationship** (e.g. studied together, co-existance, homology, etc.)

- gp130 either homodimerizes in response to IL 6 and IL 11 or forms heterodimers with the leukemia inhibitory factor LIF receptor  **LIFR**  in response to LIF, oncostatin M  [ **OSM** ]   **OSM** , ciliary neurotrophic factor CNTF, cardiotrophin 1 CT 1 or cardiotrophin like cytokine resulting in the onset of cytoplasmic tyrosine phosphorylation cascades.  Ref: 12047380 Eur J Biochem, 2002
- Continuous treatment with Dex 10 8 mol L inhibited the endogenous production of IL 6, LIF,  **OSM** , IL 11R, and OSMR, but had no detectable effect on the expression of IL 11, CT 1, CNTF, CNTFR,  **LIFR** , or gp130.  Ref: 12110437 Bone, 2002
- Its mechanism of action is via specific binding to gp130 and either the leukaemia inhibitory factor receptor  **LIFR**  or oncostatin M  [ **OSM** ]  receptor OSMR systems at the cell surface to form an active signalling complex.  Ref: 10997905 Structure Fold Des, 2000
- The expression of all other cytokines LIF, IL 11, CNTF and receptors IL 6 R, LIF R  [ **LIFR** ] , IL 11 R,  **OSM**  R, CNTF R was found in different patterns in the adenoma subtypes and normal pituitaries.  Ref: 11095488 J Clin Endocrinol Metab, 2000
- Using RT PCR we detected transcripts of the receptor components involved in  **OSM**  signaling, gp130,  **OSM**  receptor OSMR beta, and leukemia inhibitory factor receptor  [ **LIFR** ]   **LIFR** , in HCEC.  Ref: 11706938 J Neuropathol Exp Neurol, 2001
- Flow cytometric analyses detected both the  **OSM**  specific receptor and the gp130 receptor at the chondrocyte cell surface, but failed to detect the leukemia inhibitory factor receptor  [ **LIFR** ]   **LIFR** .  Ref: 11465713 Arthritis Rheum, 2001
- The Y759 motif in gp130 also attenuates gene induction mediated by the oncostatin M  [ **OSM** ]  and leukemia inhibitory factor receptor  [ **LIFR** ]  complexes, which both contain gp130 as the shared subunit.  Ref: 10946280 J Immunol, 2000
- We observed the expression of mRNA transcripts for  **OSM** ,  **OSM**  receptor subunit beta, leukaemia inhibitory factor receptor subunit  **LIFR** , and glycoprotein 130 in endometrial and endometriotic stromal cells.  Ref: 11420390 Mol Hum Reprod, 2001
- Since LIF together with LIFRalpha appear to form a more stable complex with gp130 than  **OSM**  with gp130 and OSMRbeta, co activation of  **LIFR**  and OSMR resulted in a predominant LIF like response.  Ref: 10854424 J Biol Chem, 2000
- All of these cell lines express the receptor components of  **OSM**  and leukemia inhibitory factor LIF gp130,  **LIFR**  and the  **OSM**  specific OSMRbeta.  Ref: 11038252 Brain Res Mol Brain Res, 2000
- gp130, the leukemia inhibitory factor receptor  [ **LIFR** ]  LIFRbeta and the  **OSM**  specific receptor OSMRbeta.  Ref: 11811789 Growth Factors, 2001
- Eighteen pituitary adenoma biopsies removed in transsphenoidal surgery six corticotrophic adenomas, four nonfunctioning adenomas, four somatotrophinomas, four prolactinomas and six normal anterior pituitaries were examined for the expression of IL 6 receptor R, leukemia inhibitory factor LIF, LIF R  [ **LIFR** ] , IL 11, IL 11 R, oncostatin M  [ **OSM** ]   **OSM** ,  **OSM**  R, ciliary neurotrophic factor CNTF, CNTF R and cardiotrophin 1 CT 1.  Ref: 11095488 J Clin Endocrinol Metab, 2000
